# Freshwater and Marine Environments in California Are a Reservoir of Carbapenem-Resistant Bacteria

**DOI:** 10.3390/microorganisms12040802

**Published:** 2024-04-16

**Authors:** Ashley McCarley, Manuel Luis Espejo, Dana E. Harmon, Cristian Ruiz

**Affiliations:** Department of Biology, California State University Northridge, Northridge, CA 91330, USA

**Keywords:** carbapenems, carbapenem-resistant bacteria, carbapenemase, *Aeromonas*, *Pseudomonas*, *Enterobacter*, *Enterococcus*, *Sphingobacterium*, *Paenibacillus*

## Abstract

Carbapenems are last-resort antibiotics used to treat multidrug-resistant bacterial infections. Resistance to carbapenems has been designated as an urgent threat and is increasing in healthcare settings. However, little is still known about the distribution and characteristics of carbapenem-resistant bacteria (CRB) outside of healthcare settings. Here, we surveyed the distribution of CRB in ten diverse freshwater and seawater environments in California, U.S., ranging from San Luis Obispo County to San Bernardino County, combining both direct isolation and enrichment approaches to increase the diversity of isolated CRB. From the locations surveyed, we selected 30 CRB for further characterization. These isolates were identified as members of the genera *Aeromonas*, *Enterobacter*, *Enterococcus*, *Paenibacillus*, *Pseudomonas*, *Sphingobacterium*, and *Stenotrophomonas*. These isolates were resistant to carbapenems, other β-lactams, and often to other antibiotics (tetracycline, gentamicin, or ciprofloxacin). We also found that nine isolates belonging to the genera *Aeromonas*, *Enterobacter* (*bla*_IMI-2_), and *Stenotrophomonas* (*bla*_L1_) produced carbapenemases. Overall, our findings indicate that sampling different types of aquatic environments and combining different isolation approaches increase the diversity of the environmental CRB obtained. Moreover, our study supports the increasingly recognized role of natural water systems as an underappreciated reservoir of bacteria resistant to carbapenems and other antibiotics, including bacteria carrying carbapenemase genes.

## 1. Introduction

Carbapenems are broad-spectrum, last-resort, β-lactam antibiotics used to treat multidrug-resistant infections [1,2,3,4]. They are used primarily to treat infectious bacteria resistant to other β-lactam antibiotics because carbapenems are resistant to hydrolysis by common β-lactamases [1,2,3,5]. Incidents related to carbapenem-resistant bacteria (CRB) have rapidly risen since the emergence of carbapenem-resistant Enterobacteriaceae (CRE) in the 1990s [6,7]. In 1990, there were almost no reported cases of CRE [8]. However, as of 2017, carbapenemase-producing CRE were found in every state of the U.S. [9].

Alongside decreased permeability and increased efflux, one of the most common forms of resistance to carbapenems is through the production of unique β-lactamases called carbapenemases, which are enzymes capable of degrading carbapenems [1,6]. Carbapenemases are found in Ambler classes A, B, and D, and are divided into two families based on their active site [10]. Class A carbapenemases contain a serine in their active site, while class B carbapenemases are metallo-enzymes that contain zinc ions [10]. Class D are also serine-based enzymes, but they are historically distinguished due to their ability to rapidly hydrolyze oxacillin [10,11]. Because carbapenems were originally found to resist hydrolysis by β-lactamases, the emergence of carbapenemases is of significant concern [10,12]. This problem is exacerbated by the fact that genes encoding for these enzymes are often found on genetic mobile elements such as conjugative plasmids [10], which favors their spread.

CRB pose a significant public health challenge. These bacteria are primarily associated with infections acquired in healthcare settings, and their prevalence in such environments is increasing [7,13,14,15,16,17,18,19,20,21,22,23,24,25,26]. In addition to CRE, which have been classified as an urgent threat by the Centers for Disease Control and Prevention (CDC), *Acinetobacter baumannii* and *Pseudomonas aeruginosa* strains that exhibit resistance to multiple antibiotics, including carbapenems, have been identified by the CDC as serious threats, often with limited treatment options [27]. However, despite their significant impact on public health and their increasing prevalence in healthcare settings, there is a notable lack of knowledge about the distribution of CRB and carbapenemase genes in the environment, particularly in the United States. Previous efforts to identify these bacteria have primarily focused on healthcare facilities or closely associated areas such as hospital wastewater [28,29,30,31,32]. However, recent research from Europe, Africa, and Asia has revealed the presence of carbapenem-resistant bacteria and genes in various environmental samples, including freshwater [33,34,35,36,37,38,39,40]. In the US, only two studies have examined CRB in water environments. First, a study conducted between 1999 and 2001 found CRB in seven out of sixteen rivers in the Midwest [41,42]. More recently, a study from our group examined the distribution and characteristics of CRB in ponds and lakes in the Los Angeles, CA area [43]. This study, together with a second study from our group investigating CRB in Southern California soils [44], has revealed that CRB and carbapenemase genes are more widespread than previously thought. However, further studies are still needed to better understand the spread, diversity, and characteristics of CRB in the environment in the US.

Here, we have investigated the frequency, distribution, and characteristics of CRB in freshwater and seawater aquatic environments in the broader region of the Central Coast and Southern California, using different isolation approaches to increase the diversity of the environmental CRB recovered. Overall, we have found CRB in all tested aquatic environments, although with variable abundance. We have also identified 30 CRB isolates, characterized their antibiotic resistance profiles, and found that all Gram-negative isolates were resistant to at least one non-carbapenem antibiotic, including seven isolates that were resistant to all but one of the antibiotics tested. We have also identified nine isolates that produce carbapenemases.

## 2. Materials and Methods

### 2.1. Collection of Water Samples and Isolation of Carbapenem-Resistant Bacteria (CRB)

**Figure 1:**
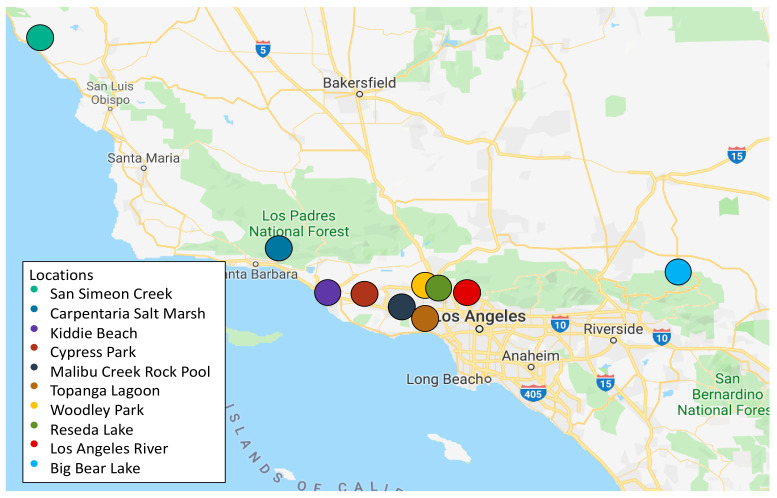
Map of the locations sampled in this study for CRB.

Between April 2018 and March 2019, we collected thirty water samples from ten different locations (Figure 1) to determine the abundance of CRB in these samples and isolate CRB for further characterization. Of the ten locations selected, seven had not been previously studied and three had been studied in our earlier experiments using a different isolation approach [43] and were resampled for comparison. The 10 locations surveyed here included a larger geographical area than our previous study and stretched from San Simeon, California to Big Bear Lake, California. Locations were selected considering their overall sanitation, proximity to residential areas, and/or proximity to agriculture. For example, Kiddie Beach in Oxnard, California is located in Ventura County not far from the Port Hueneme Naval Base. This particular beach is part of a sub-watershed for the western portion of the city of Oxnard, which includes the Oxnard west drain, residential runoff from both housing and the harbor, and runoff from the naval base [45]. In the years prior to sampling, the beach failed health and sanitation checks due to an overabundance of bacteria, as well as high levels of toxic metals such as zinc and lead [45,46]. While the outbreak has been controlled, bacterial levels were on the rise again in March 2018 [47], making it a prime sampling location. Likewise, all other locations were sampled based on their environmental health reports and history of pollution.

For each location, four liters of surface water were collected in sterile vessels that were instantly closed and then transported to the lab immediately for testing under aseptic conditions. From each sample, 100 μL of water were directly plated into MacConkey agar (Fisher Scientific, Hampton, NH, USA) to determine the total count of Gram-negative bacteria, or MacConkey agar supplemented with 2 μg/mL meropenem (Ark Pharm, Inc., Arlington Heights, IL, USA) (MAC + M2) to determine the count of carbapenem-resistant Gram-negative bacteria. In addition, another 10 μL of water were spot-plated in MacConkey and MAC + M2 media after undergoing serial dilutions from 10^0^ to 10^−4^ to determine total and CRB counts in samples with large bacterial counts. The plates were then grown at 37 °C for 18–24 h in aerobic conditions. Meropenem was the carbapenem used for the selection of CRB due to its greater effectiveness against Gram-negative bacteria [1], and because these bacteria are the main concern in healthcare settings. The concentration of meropenem used was the equivalent of “Intermediate” (or half the concentration for Resistant) of the Clinical and Laboratory Standards Institute (CLSI) minimum inhibitory concentration (MIC) break-point (4 μg/mL) for Enterobacteriaceae [48]. This concentration was selected to maximize the isolation of environmental CRB, especially when using a growth medium that is highly selective such as the MacConkey medium. It was also half of the concentration used in our previous study [43].

After initial collection and plating, the rest of the water sample was split into two-liter aliquots, each of which was filtered using a Stericup and Steritop vacuum-driven disposable bottle-top filter with a size of 0.22 μm, in order to concentrate the bacteria present in each aliquot. One filter was plated directly onto MacConkey agar supplemented with meropenem at 2 μg/mL and the other filter was placed into a BLCVM9 broth supplemented with 2 μg/mL of meropenem. Both the broth and the plate were grown at 37 °C for 24 h in aerobic conditions. BLCVM9 broth was developed for this study and contains 0.15% bile salts, 1% lactose, 0.001% crystal violet, and 1× M9 salts (Fisher Scientific). It was designed to primarily enrich enteric and Gram-negative bacteria while limiting the growth of *Stenotrophomonas maltophilia*. This bacterium is a non-lactose-fermenter and was over-represented in our previous study [43] because it is an abundant environmental opportunistic pathogen that is usually carbapenem-resistant [49]. After enrichment in BLCVM9 broth, 10 μL of culture were transferred to a MAC + M2 plate, and another 10 μL to a Mueller–Hinton agar plate supplemented with 2 μg/mL of meropenem and 40 μg/mL of X-gal (5-bromo-4-chloro-3-indolyl-β-D-glucopyranoside; to identify lactose-fermenters) (MH + X-gal + M2) to be struck for isolation. Representative colonies were restruck on MAC + M2 or MH + X-gal + M2, respectively, to confirm them as CRB, prioritizing lactose fermenters (colonies that were pink on MAC + M2 or blue on MH + X-gal + M2) when identified.

### 2.2. Selection, Identification, and Characterization of CRB Isolates

Among all isolated colonies obtained for each sample and isolation approach, we selected for further characterization only representative colonies that were phenotypically distinct to avoid selecting duplicates of the same isolate type from the same sample. Therefore, the number of selected isolates of each genus is not proportionally representative of their abundance in each sample. Overall, we selected a total of 30 distinct CRB from the 10 samples analyzed using these criteria.

The 30 CRB isolates selected for further characterization were first identified by Gram staining and PCR amplification of their 16S rRNA genes, followed by Sanger sequencing and BLAST analysis as previously described by Harmon et al. [43]. The oxidase test was performed as previously described [43] to further distinguish between closely related *S. maltophilia*, which is oxidase negative, and *Pseudomonas* species, most of which are oxidase positive [50]. After initial identification by 16S rRNA gene sequencing and BLAST analysis, the 16S sequences obtained were further analyzed by constructing phylogenetic trees based on the genus of the isolate, as determined by BLAST [51], and other sequences for the same genus found in GenBank (https://www.ncbi.nlm.nih.gov/genbank/ accessed on 13 April 2024). All of the sequences were aligned using ClustalW (https://www.genome.jp/tools-bin/clustalw accessed on 13 April 2024) [52] pairwise alignment first, and then ClustalW multiple alignment, both with the parameters set to a Gap open penalty of 15 and a Gap extension penalty of 6.66, and with the Weight Matrix set to IUB. The phylogenetic trees were constructed using Mega7 software (v7.0.26) [53], the Neighbor-Joining method, and the Jukes–Cantor statistical method, using 500 Bootstraps per tree.

The antibiotic susceptibility profile for each CRB isolate was determined using the Kirby–Bauer method [54] and the reference strain *E. coli* ATCC 25922 as a quality control, as we have previously described [43], and using three replicates per isolate and antibiotic. Gram-negative bacteria were tested using meropenem (10 μg), imipenem (10 μg), amoxicillin/clavulanic acid (20 μg/10 μg), cefotaxime (30 μg), ciprofloxacin (5 μg), gentamicin (10 μg), and tetracycline (30 μg) disks. The disks tested for Gram-positive bacteria included carbapenems: meropenem (10 μg), imipenem (10 μg), ertapenem (10 μg), and doripenem (10 μg); other β-lactams: amoxicillin/clavulanic acid (20 μg/10 μg); ampicillin (10 μg); vancomycin (30 μg); ciprofloxacin (5 μg), and tetracycline (30 μg) disks. For the vancomycin test, plates were incubated for a complete 24 h as recommended by the CLSI [48]. All antibiotic disks were purchased from Becton Dickinson (Franklin Lakes, NJ, USA). To determine whether isolates were susceptible, intermediate, or resistant to an antibiotic, we compared the average zone of inhibition diameter measurements for each isolate with the CLSI zone diameter breakpoint values [48] when available, or the European Committee on Antimicrobial Susceptibility Testing (EUCAST) values [55] as an alternative. For taxa in which the zone diameter breakpoints were not provided by CLSI or EUCAST, we used the CLSI Enterobacteriaceae breakpoint values [48].

### 2.3. Identification of Carbapenemase-Producing CRB Isolates

To identify carbapenemase-producing CRB isolates, we utilized the CarbaNP assay as previously described [43,44] to detect carbapenem hydrolysis. Briefly, we performed the CarbaNP assay [56,57,58] following the CLSI guidelines [48] and using 6 mg/mL of meropenem. CarbaNP-positive isolates were then confirmed using the Carbapenem Inactivation Method (mCIM) [59] in accordance with CLSI guidelines [48]. For the CarbaNP test, isolates positive for carbapenemase production appear as yellow because hydrolysis of meropenem lowers the pH and changes the color of the phenol red indicator [56,57,58]. Confirmation of carbapenemase production using the mCIM method involved observing a zone of inhibition of between 6 and 15 mm for *E. coli* ATCC 25922 when grown in the presence of a meropenem disk previously incubated for 4 h with the isolate being tested.

For CRB isolates confirmed as carbapenemase producers by the mCIM method, we used the EDTA-Carbapenem Inactivation Method (eCIM) to determine if the detected carbapenemase was a metallo-β-lactamase [48]. This method is similar to the mCIM, with the caveat of adding EDTA to the isolate-meropenem disk co-culture prior to the 4 h incubation to chelate metal ions and inactivate metallo-β-lactamases. As a result, metallo-β-lactamase carbapenemase producers no longer inactivate meropenem. Confirmation of metallo-β-lactamase production involved observing a zone of inhibition that increased by more than 5 mm compared to that obtained in the mCIM test.

### 2.4. Identification of Carbapenemase Genes

PCR detection of the IMI-2 carbapenemase gene (*bla*_IMI-2_) in carbapenemase-producing *Enterobacter* isolates was performed using the primers and program described by Harmon et al. [43]. PCR detection of the L1 carbapenemase gene (*bla*_L1_) in carbapenemase-producing *Stenotrophomonas* sp. isolates was performed using the primers and conditions described by Henriques et al. [40] to amplify *bla*_L1_ as previously described [43]. For each PCR, DNA-grade water was used as a non-template control, *E. coli* BW25113 as a negative control, and strains with each *bla* gene as positive controls as previously described [43].

*Aeromonas veronii* has been found to be resistant to carbapenems by producing the CphA, ImiS, or VIM-2 metallo-β-lactamases [33,60,61,62,63,64,65,66]. Thus, the following specific primers were used to detect the genes for these carbapenemases in our *A. veronii* CRB isolates. For *bla*_CphA_, the *cphA* Forward (5′-GGA TGA AGT GTG GAT TGG CCG-3′) and *cphA* Reverse (5′-TTA TGA CTG GGG TGC GGC-3′), which amplify 752 of 765 bp of the *bla*_CphA_ gene (X57102), were designed. For *bla*_ImiS_, the primers *imiS* Forward (5′-ATG ATG AAG GGT TGG ATA AAG T-3′) and *imiS* Reverse (5′-TTA TGA TTG TGA AGC CGC CT-3’) were designed to amplify 786 out of 922 bp of the *bla*_ImiS_ gene (NG_050415). For *bla*_VIM-2_, the primers designed by Belotti et al. [67] *vim-2* Forward (5′-GAT GGT GTT TGG TCG CAT A-3′) and *vim-2* Reverse (5′-CGA ATG CGC AGC ACC AG-3′) were used to amplify 390 out of the 801 bp of the *bla*_VIM-2_ gene. The PCR reaction mixes had a total volume of 50 μL per isolate, consisting of DreamTaq buffer, 0.2 mM dNTPs, 0.5 μM of each primer, 1.25 units of DreamTaq Polymerase, and 5 μL of template DNA (1 colony resuspended in 50 μL of DNA-grade water). The thermocycler programs used were: (1) for *bla*_CphA_, one cycle of 95 °C for 5 min; 35 cycles of 95 °C for 30 s, 58 °C for 40 s, and 72 °C for 50 s; one cycle of 72 °C for 7 min, and finally 4 °C for infinite; (2) for *bla*_ImiS_, one cycle of 95 °C for 5 min; 35 cycles of 95 °C for 30 s, 53 °C for 30 s, and 72 °C for 60 s; one cycle of 72 °C for 10 min; and finally 4 °C for infinite; (3) for *bla*_VIM-2_, one cycle of 95 °C for 10 min; 35 cycles of 95 °C for 30 s, 52 °C for 40 s, and 72 °C for 50 s; then 72 °C for 5 min; and 4 °C for infinite time.

All carbapenemase gene PCR products were visualized by electrophoresis in a 1% agarose gel supplemented with 10 μL of 1:10,000 ethidium bromide prior to their submission to Laragen Inc. (Culver City, CA, USA) for Sanger sequencing. The sequences obtained were analyzed using LALIGN (SIB ExPASy: https://embnet.vital-it.ch/software/LALIGN_form.html accessed on 13 April 2024) to compare and align our sequences to reference sequences *bla*_L1_ (GenBank Accession number NG_047502) and *bla*_IMI-2_ (GenBank Accession number DQ173429).

## 3. Results and Discussion

### 3.1. Distribution, Abundance, and Isolation of Carbapenem-Resistant Bacteria (CRB) in Diverse Aquatic Environments

The rise of CRB in healthcare settings during the past 30 years is a global threat [6,7,13,14,15,16,17,18,19,20,21,22,23,24,25,26]. However, the role that the environment plays as a reservoir for CRB is not entirely understood, especially in the United States. In a previous study, we have identified CRB in a limited-scope survey of ponds and lakes in the Los Angeles (CA) area [43]. Therefore, our first goal here was to expand this survey to include a larger geographical area in South and Central California (ranging from San Simeon Creek in the north to Big Bear Lake in the east), and other types of aquatic environments such as rivers, marshes, estuaries, and beaches (Figure 1 and Table 1). The 10 locations sampled were selected because of their proximity to the community around them, long-standing history with pollution, or a history of bacterial outbreaks. Overall, we detected CRB in all aquatic environments tested, although with a low abundance (<10 to 20 CFU/mL), except for the Carpinteria Salt Marsh (5.2 × 10^3^ CFU/mL, which is similar to the total bacterial counts obtained for this sample; Table 1). We speculate that the Thomas fire in December 2017, followed by a rain mudslide in January 2018, contributed to the higher total and CRB counts in this location. For the rest of the sampled locations, the low abundance of CRB found in this study in most samples is comparable to those in our previous study [43], and those previously found in Portuguese rivers [33].

**Figure 2:**
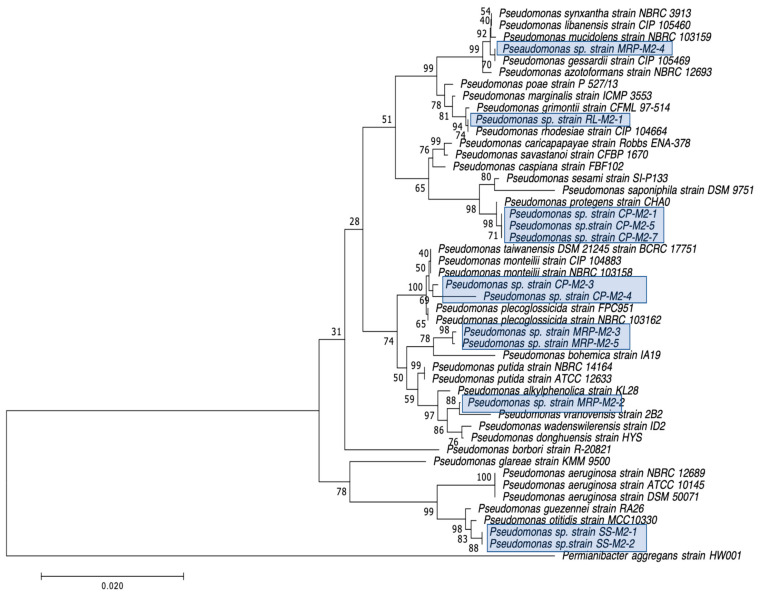
Phylogenetic tree showing the relatedness between the 16S rRNA gene sequences of the carbapenem-resistant *Pseudomonas* sp. isolates obtained in this study (indicated in blue boxes) and those from *Pseudomonas* sp. isolates from previous studies obtained from GenBank. The scale bar at the bottom of the tree represents the number of nucleotide substitutions per site.

**Figure 3:**
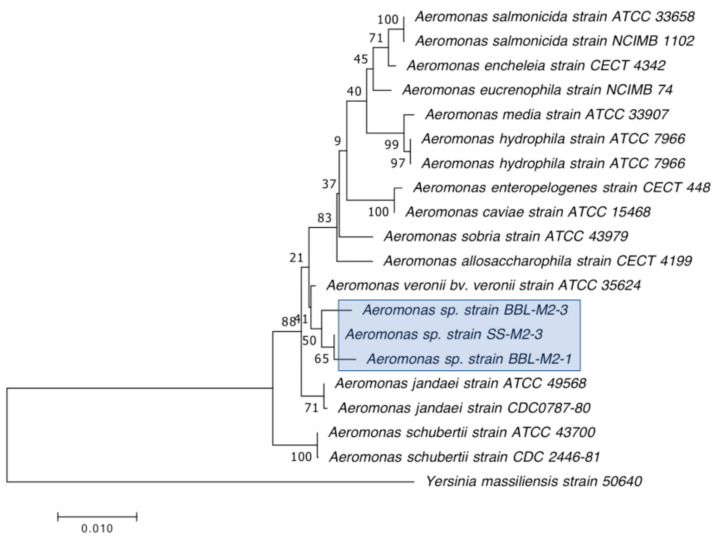
Phylogenetic tree showing the relatedness between the 16S rRNA gene sequences of the carbapenem-resistant *Aeromonas* sp. isolates obtained in this study (indicated in blue boxes) and those from *Aeromonas* sp. isolates from previous studies obtained from GenBank. The scale bar at the bottom of the tree represents the number of nucleotide substitutions per site.

**Figure 4:**
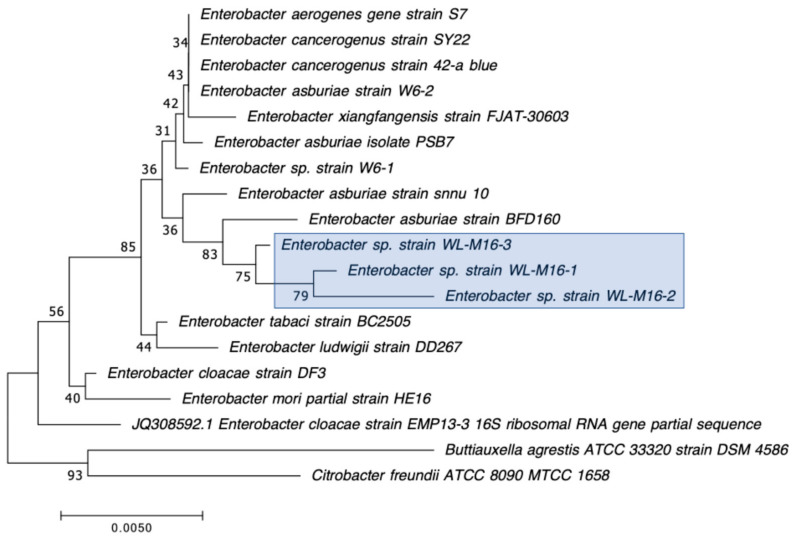
Phylogenetic tree showing the relatedness between the 16S rRNA gene sequences of the carbapenem-resistant *Enterobacter* sp. isolates obtained in this study (indicated in blue boxes) and those from *Enterobacter* sp. isolates from previous studies obtained from GenBank. The scale bar at the bottom of the tree represents the number of nucleotide substitutions per site.

**Figure 5:**
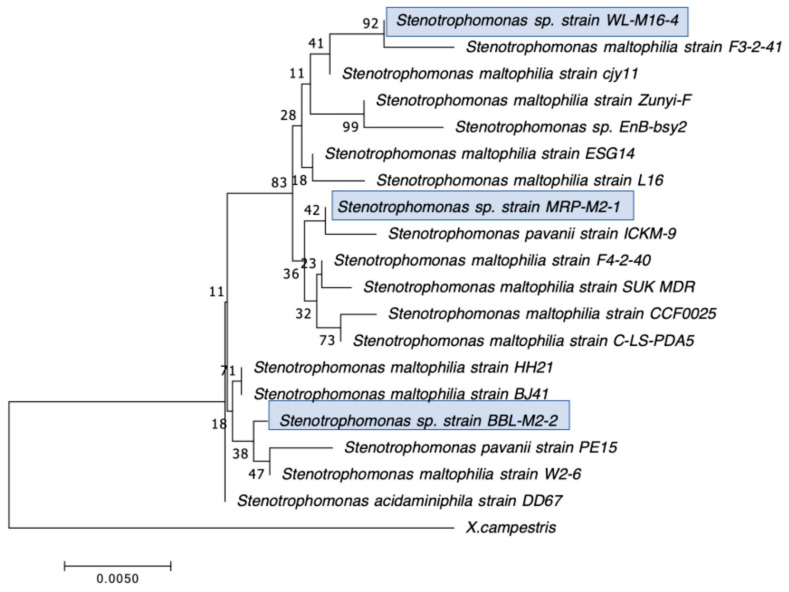
Phylogenetic tree showing the relatedness between the 16S rRNA gene sequences of the carbapenem-resistant *Stenotrophomonas* sp. isolates obtained in this study (indicated in blue boxes) and those from *Stenotrophomonas* sp. isolates from previous studies obtained from GenBank. The scale bar at the bottom of the tree represents the number of nucleotide substitutions per site.

**Figure 6:**
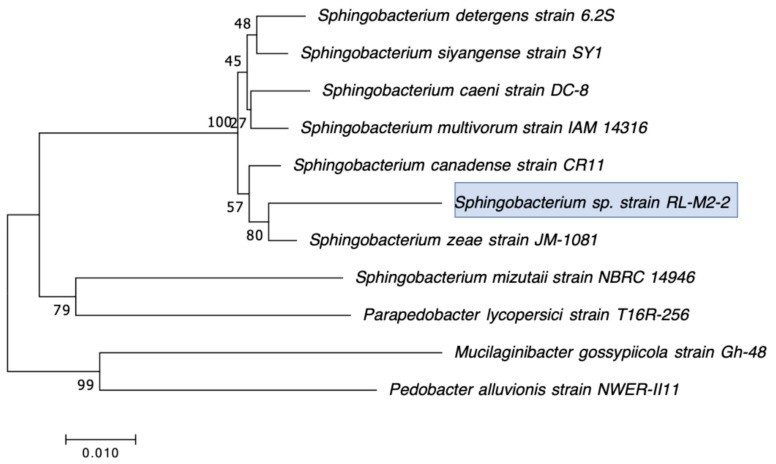
Phylogenetic tree showing the relatedness between the 16S rRNA gene sequences of the carbapenem-resistant *Sphingobacterium* sp. isolates obtained in this study (indicated in blue boxes) and those from *Sphingobacterium* sp. isolates from previous studies obtained from GenBank. The scale bar at the bottom of the tree represents the number of nucleotide substitutions per site.

**Figure 7:**
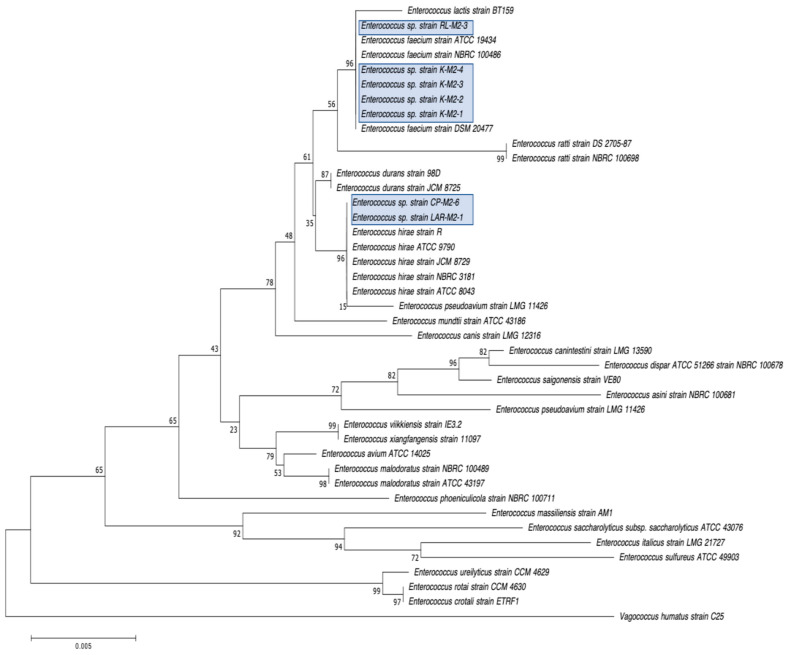
Phylogenetic tree showing the relatedness between the 16S rRNA gene sequences of the carbapenem-resistant *Enterococcus* sp. isolates obtained in this study (indicated in blue boxes) and those from *Enterococcus* sp. isolates previous studies obtained from GenBank. The scale bar at the bottom of the tree represents the number of nucleotide substitutions per site.

**Figure 8:**
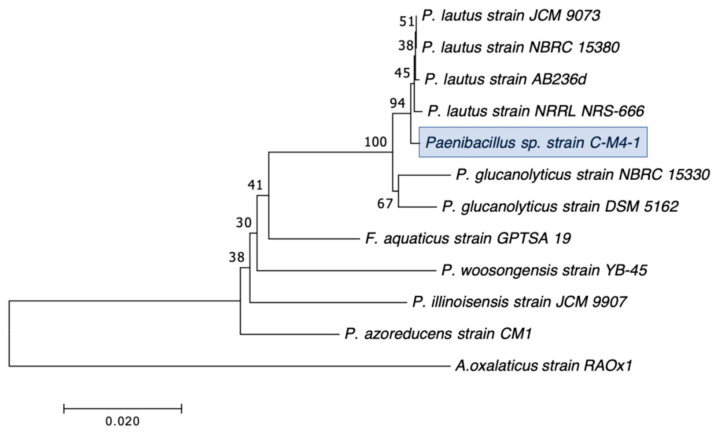
Phylogenetic tree showing the relatedness between the 16S rRNA gene sequences of the carbapenem-resistant *Paenibacillus* sp. isolates obtained in this study (indicated in blue boxes) and those from *Paenibacillus* sp. isolates previous studies obtained from GenBank. The scale bar at the bottom of the tree represents the number of nucleotide substitutions per site.

Our second goal was to gain a deeper understanding of the diversity of CRB present in these environments. In our previous study, we had used MacConkey with 4 μg/mL meropenem media as our primary isolation media, and found that all CRB isolates were Gram-negatives, especially *Stenotrophomonas* sp. (63% of the isolates) and *Pseudomonas* sp. (22% of the isolates), followed by *Enterobacter* sp., *Aeromonas* sp., and *Cupriavidus* sp. isolates [43]. In the present study, we employed MacConkey agar with 2 μg/mL meropenem, a less stringent concentration of carbapenem, and added a second isolation approach based on first enriching CRB using BLCVM9 broth supplemented with 2 μg/mL of meropenem prior to streaking samples in both MacConkey with 2 μg/mL meropenem and Mueller–Hinton with 2 μg/mL meropenem. Using this approach, only three isolates (10%) out of the thirty CRB characterized were *Stenotrophomonas* sp., and new CRB types were identified, including Gram-positives (*Enterococcus* sp. and *Paenibaciullus* sp.) and *Sphingobacterium* sp. isolates (Figure 2, Figure 3, Figure 4, Figure 5, Figure 6, Figure 7 and Figure 8). These results indicate that, besides sampling different types of aquatic environments, the additional isolation approaches employed in this study may have increased the diversity of CRB obtained by limiting *Stenotrophomonas* sp. that potentially outgrew and/or outcompeted other bacteria in our previous study. This interpretation is strongly supported by the fact that seven out of the eight Gram-positive CRB isolates characterized in this study were obtained using our novel enrichment approach.

We preliminarily identified our 30 selected CRB isolates as three *Aeromonas veronii*, three *Enterobacter asburiae*, five *Pseudomonas* spp., one *Pseudomonas plecoglossicida*, three *Pseudomonas putida*, one *Pseudomonas fluorescens*, one *Pseudomonas rhodesiae*, one *Sphingobacterium siyangensis*, three *Stenotrophomonas maltophilia*, two *Enterococcus lactis*, two *Enterococcus hirae*, three *Enterococcus faecium,* and one *Paenibacillus lautus* (Figure 2, Figure 3, Figure 4, Figure 5, Figure 6, Figure 7 and Figure 8). *Pseudomomas* sp. isolates were the most abundant and widespread, comprising 12 (40%) of the 30 isolates. Prevalence of *Pseudomonas* sp. is expected from aquatic environments because *Pseudomonas* spp. are ubiquitous in the environment, being abundant in water, soil, and on plants [68]. *Stenotrophomonas* sp. is also widespread around the world, being isolated from numerous water sources [49]. Carbapenem-resistant *E. asburiae* have been previously found in aquatic environments, including one river in Portugal [33], four rivers in the midwestern US [41], and also in our previous study in a sample from Woodley Lake in Los Angeles, CA [43]. Other Gram-negatives identified in the present study included *Aeromonas veronii* and *Sphingobacterium syangensis. Aeromonas veronii* is a common aquatic bacterium that can exhibit intrinsic resistance to carbapenems [61]. In contrast, *Sphingobacterium* sp. resistant to carbapenems have not been previously reported in water environments in the U.S. to our knowledge. *Sphingobacterium syangensis* is isolated predominantly from soil [69], and members of the genus *Sphingobacterium* have been associated with pulmonary infections in cystic fibrosis patients [70]. Different species of *Sphingobacterium* sp. have been shown to possess varying levels of resistance to β-lactams (including carbapenems) and aminoglycosides [70].

Regarding the Gram-positive CRB isolated in this study, seven out of eight were *Enterococcus* sp., and one isolate was preliminarily identified as *Paenibacillus lautus* (Figure 7 and Figure 8). Interestingly, no Gram-positives were identified in our previous water study [43], although we found *Enterococcus* species in our survey for CRB in soil [44], which usually has a larger bacterial load. Our findings in this study suggest that the broader type of ecosystems sampled here, and especially the addition of an enrichment step (seven out of the eight Gram-positive CRB were obtained from the sub-samples subject to enrichment), contributed to the better isolation of Gram-positive CRB. Out of these, *Enterococcus faecium* (three isolates), all from Kiddie Beach in Oxnard, CA, was the most abundant. *E. faecium* is a human commensal organism associated with the gut microbiota [71], and thus its presence might indicate recent fecal contamination in this location. However, enterococci are also commonly found in marine environments, especially on sandy beaches due to their ability to form biofilms [71]. In recent years, enterococci, especially *Enterococcus faecium*, have been associated with nosocomial infections, including urinary tract infections, endocarditis, and bacteremia [72]. Thus, the isolation of carbapenem-resistant *E. faecium* in Kiddie Beach is a public health concern. We also found two *Enterococcus hirae* and two *Enterococcus lactis*. Interestingly, *E. hirae* has the ability to reduce copper toxicity in the environment, which is essential given the widespread use of copper in agricultural and urban settings [73]. *E. lactis* is associated with the food industry because it produces other lactic organic acids that act as biological preservatives [74]. However, *Enterococcus lactis* is not generally associated with environmental isolation, especially in aquatic environments [74]. Lastly, *Paenibacillus lautus* has been associated with both environmental and nosocomial isolation [75,76]. In the environment, *P. lautus* is considered an opportunistic pathogen isolated from the gut microbiota of ticks that can be transmitted to humans through tick bites, causing bacteremia [75]. In clinical settings, *P. lautus* was isolated from blood and abscesses in a hospital in Madrid, suggesting that it can be a nosocomial opportunistic pathogen [76].

### 3.2. Characterization of the Antibiotic Susceptibility Profile of Carbapenem-Resistant Bacteria (CRB) from Diverse Aquatic Environments

**Figure 9:**
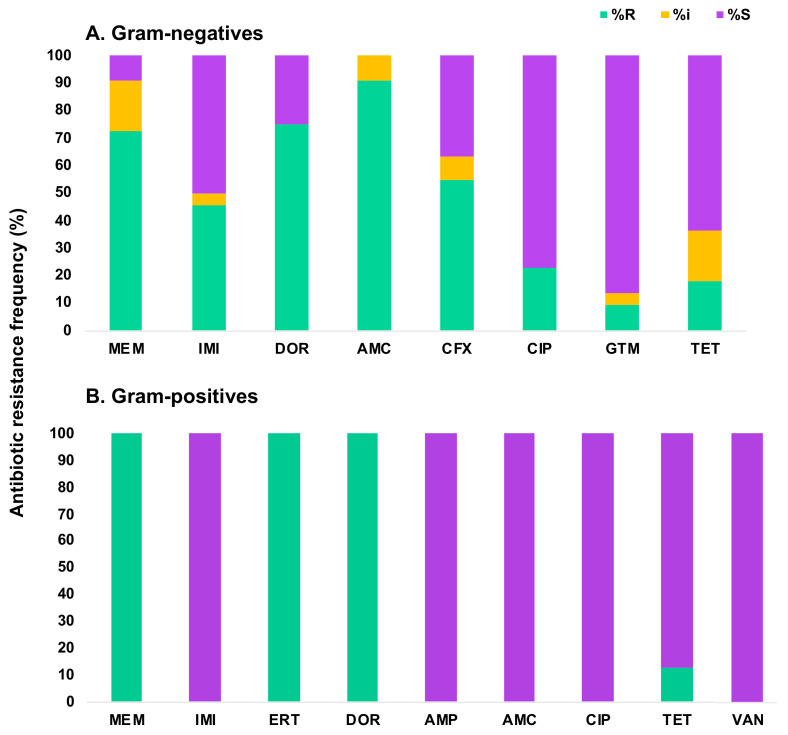
Antibiotic resistance frequency of Gram-negative (**A**) and Gram-positive (**B**) water isolates from this study. Green (% Resistant), Yellow (% Intermediate), Purple (% Susceptible). Carbapenems: meropenem (MEM), imipenem (IMI), doripenem (DOR), and ertapenem (ERT). Other β-lactams: amoxicillin-clavulanic acid (AMC), cefotaxime (CFX), and ampicillin (AMP). Other antibiotics: ciprofloxacin (CIP), gentamicin (GTM), tetracycline (TET), and vancomycin (VAN).

We next characterized the antibiotic susceptibility profiles of the 30 CRB isolates identified in this study (Table 2 and Table 3; Figure 9). Gram-negative bacteria testing included two carbapenems (meropenem and imipenem) and five non-carbapenem antibiotics (amoxicillin plus clavulanic acid, cefotaxime, ciprofloxacin, gentamicin, and tetracycline). These antibiotics were selected because they are commonly used to treat Gram-negative pathogens [48]. Overall, 20 out of the 22 selected Gram-negative CRB from all sampled locations were resistant or intermediate to at least one carbapenem, whereas two *Aeromonas* sp. isolates had zones of inhibition that were slightly above the intermediate threshold using the Enterobacteriaceae cutoffs for the carbapenems tested, and thus were scored as sensitive to carbapenems. However, both isolates would have been scored as intermediate for meropenem if we had used the *Pseudomonas* sp. EUCAST cut-off values [55]. Of the Gram-negative isolates characterized, 72.7% were resistant and 18.2% were intermediate to meropenem. In contrast, 45.5% were resistant to imipenem, predominately from the genera *Stenotrophomonas* and *Enterobacter*, and 4.5% were intermediate. Lastly, doripenem was tested against all *Pseudomonas* sp. isolates because it has shown potent inhibitory effects against *P. aeruginosa* [77]. We found that 75% of our *Pseudomonas* sp. isolates were resistant to this carbapenem. Of the non-carbapenem antibiotics, 90.9% of isolates showed resistance to amoxicillin plus clavulanic acid, and 9.1% were intermediate. Cefotaxime resistance was found in 54.5% of isolates, and 9.1% were intermediate. The isolates resistant to cefotaxime were identified as either *Pseudomonas* or *Stenotrophomonas* species. Only five isolates (all *Pseudomonas* sp., representing 22.7% of the tested Gram-negative CRB) were resistant to ciprofloxacin. For gentamicin, only 13.6% of the Gram-negative isolates tested, all of them *Stenotrophomonas* sp., were resistant or intermediate. Finally, 36.4% of the Gram-negatives CRB tested, all belonging to either the *Pseudomonas* or *Stenotrophomonas* genera, were resistant or intermediate to tetracycline (Table 2; Figure 9A). Interestingly, about one-third of the Gram-negative CRB characterized displayed a broad and concerning multidrug-resistant phenotype, being resistant or intermediate to all but one of the antibiotics tested.

All eight Gram-positive CRB isolates were tested for resistance to carbapenems and other antibiotics commonly used to treat Gram-positive pathogens [48]. These antibiotics included four carbapenems (meropenem, imipenem, ertapenem, and doripenem), and four more non-carbapenem antibiotics (amoxicillin plus clavulanic acid, ciprofloxacin, tetracycline, and vancomycin). Overall, 100% of these isolates were resistant to three out of the four carbapenems tested (meropenem, ertapenem, and doripenem) but susceptible to imipenem (which is common for Gram-positives [78]). Interestingly, all isolates were susceptible to the non-carbapenem antibiotics tested except for one *Enterococcus hirae* isolate from Cypress Park, CA that was resistant to tetracycline (Table 3; Figure 9B).

Overall, these findings indicate that aquatic environments are an important reservoir of bacteria resistant to carbapenems and other antibiotics, especially Gram-negative CRB resistant to a broad spectrum of β-lactams. Of special concern was the finding that about one-third of the Gram-negative CRB characterized were resistant to nearly all antibiotics tested, given that some of them are opportunistic pathogens or could serve as reservoirs in the transmission of antibiotic resistance determinants.

### 3.3. Detection of Carbapenemase Production and Carbapenemase Genes in CRB Isolates

After characterizing them, we used the CarbaNP assay to determine whether carbapenemases contributed to the carbapenem-resistance phenotype of the 30 selected CRB isolates. Of them, nine were CarbaNP positive: three *Enterobacter asburiae*, three *Stenotrophomonas maltophilia*, and three *Aeromonas veronii* (Table 2 and Table 4). Using PCR and sequencing to identify their carbapenemase genes, we found that the *E. asburiae* and *S. maltophilia* isolates carried the *bla*_IMI-2_ and *bla*_L1_ carbapenemase genes, respectively. These two carbapenemase genes have previously been found in *E. asburiae* plasmids or in the chromosome of *S. maltophilia* isolates, respectively [42,79]. The high identity (>98%) between the *bla*_IMI-2_ variants identified here and the *bla*_IMI-2_ reference sequence (DQ173429) is similar to that found for *E. asburiae* isolates in our previous study [43]. For the *bla*_L1_ gene, the percent DNA identity between the *bla*_L1_ variants of the isolates from this study and the reference sequence (NG_047502) was 87.7% to 88.8% (96–97% protein similarity), in accordance with our previous study [43] and the *bla*_L1_ variability previously found in *Stenotrophomonas* sp. from healthcare settings [79].

Interestingly, all three *Aeromonas veronii* isolates were positive for carbapenemase production using the CarbaNP test, despite two of them being susceptible to carbapenems according to the Enterobacteriaceae cut-offs (but intermediate to meropenem according to the *Pseudomonas* spp. cut-off) (Table 2 and Table 4). Moreover, prior studies have shown that carbapenemase-producing *Aeromonas* do not always appear as carbapenem-resistant under standard antibiotic susceptibility testing [61]. Thus, the carbapenemase activity of these three *Aeromonas* sp. isolates was further studied using the mCIM test. This test is recommended by the CLSI to confirm carbapenemase activity because the CarbaNP assay can yield false positives [59]. All three *Aeromonas* sp. isolates were confirmed as carbapenemase-positive using the mCIM test. Next, using the eCIM test [48], we found that their carbapenemases were Class B metallo-β-lactamases (Table 4). Because metallo-carbapenemase-producing *A. veronii* typically produce the chromosomal CphA, ImiS, or VIM-2 carbapenemases [61], we used PCR to test for the presence of the *bla*_CphA_, *bla*_ImiS_, or *bla*_VIM-2_ genes in our isolates. Interestingly, all three isolates tested negative for these genes, including the highly carbapenem-resistant *A. veronii* SS-M2-3 isolate. These findings indicate that these *A. veronii* isolates either may carry poorly conserved variants of these genes, or different, potentially undiscovered carbapenemase genes, which we will investigate in future studies.

## 4. Conclusions

Carbapenem-resistant bacteria (CRB) and carbapenemase genes represent a major and increasing public health challenge. However, little is known about their distribution and diversity in the environment, especially in the U.S. This study contributes to addressing this gap in knowledge by sampling diverse freshwater and seawater aquatic environments and combining both direct isolation and enrichment approaches to determine the abundance, distribution, and diversity of CRB in California, U.S. Overall, we found a low abundance of CRB in the ten locations sampled, except for the Carpinteria Salt Marsh, which had been affected by a fire followed by rains and a mudslide. Identification and characterization of 30 selected CRB from the aquatic environments sampled revealed a greater diversity of both Gram-negative and Gram-positive CRB genera, compared to a prior study focused only on freshwater environments and not including CRB enrichment. The CRB isolated in the present study belonged to the genera *Aeromonas*, *Enterobacter*, *Enterococcus*, *Paenibacillus*, *Pseudomonas*, *Sphingobacterium*, and *Stenotrophomonas*. Interestingly, we found that all Gram-negative CRB characterized in this study were also resistant or intermediate to at least one non-carbapenem antibiotic, especially other β-lactams, and that one-third of them were resistant to nearly all antibiotics tested, which is of great concern. Finally, we found that nine *Aeromonas* sp., *Enterobacter* sp. (*bla*_IMI-2_), and *Stenotrophomonas* sp. (*bla*_L1_) isolates were carbapenemase producers. Overall, these findings expand our understanding of the role of natural water environments as important and often underappreciated reservoirs of bacteria resistant to carbapenems and other antibiotics, including carbapenemase-producing bacteria.

## Figures and Tables

**Table 1:** Summary of locations sampled in this study and their total bacterial and CRB counts.

Sample	Location	Collection Date (D/M/Y)	Location Type	Coordinates	Total Bacteria (CFU/mL)	CRB (CFU/mL)
C	Carpinteria Salt Marsh ^a^	24/4/18	Marine estuary	34.401244, 119.5401337	5.1 × 10^3^	5.2 × 10^3^
K	Kiddie Beach ^b^	7/5/18	Beach harbor	34.1601144, 119.2235778	≤10	≤10
LAR	LA River ^c^	4/6/18	Urban river	34.156572, 118.291143	2.2 × 10^3^	≤10
CP	Cypress Park ^d^	18/6/18	Natural creek	34.167243, 118.962229	5.2 × 10^2^	≤10
SS	San Simeon Creek ^e^	24/6/18	Coastal wetlands	35.5971832, 121.1218039	1 × 10^2^	≤10
TL	Topanga Lagoon ^f^	23/7/18	Natural pool	34.038536, 118.583085	7 × 10^2^	≤10
BBL	Big Bear Lake ^g^	21/8/18	Natural freshwater lake	34.245278, 116.917086	8 × 10^1^	≤10
WL	Woodley Lake ^h^	18/3/19	Reclaimed water	34.175326, 118.472829	3.1 × 10^3^	10
RL	Reseda Lake ^i^	18/3/19	Artificial lake	34.188714, 118.534383	2.4 × 10^2^	20
MRP	Malibu Rock Pool ^j^	28/3/19	Natural pool	34.096555, 118.729879	1.1 × 10^2^	≤10

^a^ Estuary fed by the Franklin, Santa Monica, and San Simeon creeks. ^b^ Kiddie Beach (Oxnard marina) receives residential and military runoff water. ^c^ The Los Angeles River runs from Simi Valley, CA to Long Beach, CA, bisecting the city. The riverbed alternates between cement and natural dirt. Our sample was collected at Glendale Narrows, which is an area of restoration where cement was removed to expose the natural riverbed. ^d^ Our sample was collected at Arroyo Conejo Creek, which spans the entire Conejo Valley and is the only watershed for the entire valley. The area is highly residential. ^e^ The creek is a natural wetland that receives agricultural and residential runoff. ^f^ Lagoon that forms behind a beach berm after seasonal rains and is fed by the Topanga Creek. ^g^ Natural reservoir for the San Bernardino Mountains, it is filled solely by snow runoff. ^h^ Wildlife wetland and bird sanctuary that is filled with reclaimed water from the Tillman Water Reclamation Plant (DCTWRP). ^i^ Asphalt lined urban lake with potable water that is treated with an algicide. ^j^ Natural pool filled with rain runoff from the Santa Monica Mountains.

**Table 2:** Gram-negative CRB water isolates identified and characterized in this study.

Isolate ^a^	Closest Species Identified by BLAST of 16S rRNA Gene	CarbaNP ^c^	CP ^d^	Inhibition Zone (Diameter in mm) ^b^							
				MEM	IMI	DOR	AMC	CFX	CIP	GTM	TET
**CP-M2-1**	*Pseudomonas* sp. 18	−	N/D	0	16.7	16.7	0	5	22.6	18.3	6
**CP-M2-3**	*Pseudomonas plecoglossicida*	−	N/D	5.3	27	19	0	10	25.3	18.3	14
**CP-M2-4**	*Pseudomonas putida*	−	N/D	0	26	14.5	0	4.3	22.7	21.3	12
**CP-M2-5**	*Pseudomonas* spp. GC04	−	N/D	0	28.3	20.8	0	3.7	26	26	12.3
**CP-M2-7**	*Pseudomonas* sp. CHZYR63	−	N/D	0	19	16.3	0	0	23.3	17	7
SS-M2-1	*Pseudomonas* sp. J4AJ	−	N/D	18.7	24.7	20.6	10	25.3	38.8	24.7	25.3
**SS-M2-2**	*Pseudomonas* sp. P7	−	N/D	9.7	27.3	18.4	5.7	25.3	33	23.7	16
SS-M2-3	*Aeromonas veronii*	+	UNK	7	8	N/D	8	35	39	25	25.3
BBL-M2-1	*Aeromonas veronii*	+	UNK	24.7	20.7	N/D	15	36.7	36.7	20.3	27.3
**BBL-M2-2**	*Stenotrophomonas maltophilia*	+	*bla*_L1_	0	0	N/D	0	0	26.7	0	11.7
BBL-M2-3	*Aeromonas veronii*	+	UNK	23.5	24.7	N/D	9	34	31.8	17.8	27
***MRP-M2-1***	*Stenotrophomonas maltophilia*	+	*bla*_L1_	0	0	N/D	0	0	26	7.3	10.7
MRP-M2-2	*Pseudomonas* sp. W15Feb40A	−	N/D	19.8	30.7	28.5	3.3	15.3	24.7	22.8	16
MRP-M2-3	*Pseudomonas putida*	−	N/D	17	29.7	27.3	0	15	31.3	23.3	19.3
MRP-M2-4	*Pseudomonas fluorescens*	−	N/D	19.7	21	21	0	4.7	35.7	32	34
MRP-M2-5	*Pseudomonas putida*	−	N/D	12	25.3	22	0	13	28.7	23.7	17.3
RL-M2-1	*Pseudomonas rhodesiae*	−	N/D	0	13.5	13	0	3	36	30	21.7
RL-M2-2	*Sphingobacterium siyangensis*	−	N/D	21.2	15.8	N/D	13.7	26	29.7	15	24.5
***WL-M2-1***	*Enterobacter asburiae*	+	*bla*_IMI-2_	2.2	0	N/D	0	32	35.7	21.3	23.3
WL-M2-2	*Enterobacter asburiae*	+	*bla*_IMI-2_	2.2	0	N/D	0	32.3	35.7	21.3	23.3
WL-M2-3	*Enterobacter asburiae*	+	*bla*_IMI-2_	2.2	0	N/D	0	31.7	36	21.7	24
WL-M2-4	*Stenotrophomonas maltophilia*	+	*bla*_L1_	0	0	N/D	0	0	26.7	13.3	10.8

^a^ Isolate number: in regular font, isolates obtained directly using MacConkey-meropenem medium; in bold, isolates enriched in BLCVM9-meropenem medium before streaking in MacConkey-meropenem medium; in bold and italics, isolates enriched in BLCVM9-meropenem medium before streaking in Mueller–Hinton-meropenem medium. The initial(s) before the first dash in the isolate designation indicates the isolation location (Table 1). ^b^ Resistant (green), Intermediate (yellow), Susceptible (purple), based on the CLSI [48] or EUCAST [55] (for *Pseudomonas* spp.) breakpoint values of the diameters of the zone of inhibitions of each antibiotic. For genera that did not have diameter breakpoint values by CLSI or EUCAST, the Enterobacteriaceae cutoff values were used. N/D indicates that it was not tested. MEM (meropenem), IMI (imipenem), DOR (doripenem), AMC (amoxicillin plus clavulanic acid), CFX (cefotaxime), CIP (Ciprofloxacin), GTM (Gentamicin), TET (Tetracycline). ^c^ “−” and “+” indicate a negative or positive result, respectively, for the CarbaNP test. ^d^ Carbapenemase gene identified by PCR and sequencing. UNK (unknown) indicates lack of detection of a carbapenemase gene by PCR using *bla*_cphA_, *bla*_ImiS_, or *bla*_VIM-2_ primers.

**Table 3:** Gram-positive CRB water isolates identified and characterized in this study.

Isolate ^a^	Closest Species Identified by BLAST of 16S rRNA Gene	CarbaNP ^c^	Inhibition Zone (Diameter in mm) ^b^								
			MEM	IMI	ERT	DOR	AMP	AMC	CIP	TET	VAN
***C-M4-1***	*Paenibacillus lautus*	−	5	25	0	12.3	23.3	26.7	22.3	34.3	26.3
***K-M2-1***	*Enterococcus lactis*	−	2.7	23.7	2.3	11.7	25.7	31.3	23.6	30	24.3
***K-M2-2***	*Enterococcus faecium*	−	2	24.3	0	9.6	21	29	24	29.3	24.8
***K-M2-3***	*Enterococcus faecium*	−	4.7	22.7	4.7	9.7	21.7	27.3	23.3	27.7	24
***K-M2-4***	*Enterococcus faecium*	−	0	20.3	0	6.7	19.3	27.3	23.3	30.7	25.3
***LAR-M2-1***	*Enterococcus hirae*	−	5.5	24.7	8.3	10.7	22	27	24	26	22.5
***CP-M2-6***	*Enterococcus hirae*	−	11	28	13	16.2	29.3	32	23	2.8	22.3
RL-M2-3	*Enterococcus lactis*	−	14	27	11.7	11.3	25.7	27.3	25	30.3	25.3

^a^ Isolate number: in regular font, isolate obtained directly on MacConkey-meropenem medium; in bold and italics, isolates enriched in BLCVM9-meropenem medium before streaking in Mueller–Hinton-meropenem medium. Intials(s) before the first dash indicates the isolation location (Table 1). ^b^ Resistant (green), Susceptible (purple), based on the CLSI [48] and EUCAST zone of inhibition diameter breakpoint values [55]. For genera that did not have diameter data in the CLSI manual, Enterobacteriaceae cut-offs were used. MEM (meropenem), IMI (imipenem), ERT (ertapenem), DOR (doripenem), AMP (ampicillin), AMC (amoxicillin plus clavulanic acid), CIP (ciprofloxacin), TET (tetracycline), VAN (vancomycin). ^c^ “−” indicate a negative result for the CarbaNP test.

**Table 4:** Detection of Carbapenemases Production by CarbaNP, mCIM, and eCIM assays, and identification of carbapenemase genes by PCR and Sequencing.

Isolate	Closest Species Identified by 16S rRNA Gene	CarbaNP	mCIM	eCIM	Carbapenemase Gene (% DNA Identity)	Carbapenemase (% Amino Acid Similarity)
SS-M2-3	*Aeromonas veronii*	+	+	+	Unknown	Unknown
BBL-M2-1	*Aeromonas veronii*	+	+	+	Unknown	Unknown
BBL-M2-2	*Stenotrophomonas maltophilia*	+	N/D	N/D	*bla*_L1_ (88.6%)	L1 (96.9%)
BBL-M2-3	*Aeromonas veronii*	+	+	+	Unknown	Unknown
MRP-M2-1	*Stenotrophomonas maltophilia*	+	N/D	N/D	*bla*_L1_ (87.7%)	L1 (96.0%)
WL-M16-1	*Enterobacter asburiae*	+	N/D	N/D	*bla*_IMI-2_ (99.2%)	IMI-2 (98.6%)
WL-M16-2	*Enterobacter asburiae*	+	N/D	N/D	*bla*_IMI-2_ (98.3%)	IMI-2 (97.7%)
WL-M16-3	*Enterobacter asburiae*	+	N/D	N/D	*bla*_IMI-2_ (99.8%)	IMI-2 (100%)
WL-M16-4	*Stenotrophomonas maltophilia*	+	N/D	N/D	*bla*_L1_ (88.2%)	L1 (97.0%)

Note: Unknown indicates the inability to identify the carbapenemase gene using targeted PCR with *bla*_CphA_-, *bla*_ImiS_-, or *bla*_VIM-2_-specific primers. N/D: Not Determined (for carbapenemases directly identified by PCR and sequencing). % DNA identify or amino acid similarity compared to the reference sequences *bla*_L1_ (NG_047502) and *bla*_IMI-2_ (DQ173429). “+” Indicates a positive result for that test.

## Data Availability

All 16S rRNA gene sequences obtained in this study have been deposited in GenBank (https://www.ncbi.nlm.nih.gov/genbank/) under the following accession numbers: MT790713–MT790742.
